# Crystal structure of poly[aqua­[μ-1,1′-(9,9-dimethyl-9*H*-fluoren-2,7-di­yl)di-1*H*-imidazole](μ-naphthalene-1,4-di­carboxyl­ato)nickel(II)]

**DOI:** 10.1107/S1600536814017681

**Published:** 2014-08-06

**Authors:** Hengye Zou, Yanjuan Qi

**Affiliations:** a Department of Chemistry, Changchun Normal University, Changchun 130032, People’s Republic of China

**Keywords:** crystal structure, nickel complex, naphthalene-1,4-di­carboxyl­ate, hydrogen bonding, double chain

## Abstract

In the title compound, [Ni(C_12_H_6_O_4_)(C_21_H_18_N_4_)(H_2_O)]_*n*_, the Ni^II^ cation is coordinated by three carboxyl­ate O atoms of two naphthalene-1,4-di­carboxyl­ate anions, one water mol­ecule and two N atoms of two 1,1′-(9,9-dimethyl-9*H*-fluoren-2,7-di­yl)di-1*H*-imidazole (DFDI) ligands, giving rise to a slightly distorted octa­hedral geometry. The Ni^II^ ions are linked by the DFDI ligands into chains, which are further connected by the carboxyl­ate anions into double chains that elongate in the the *b*-axis direction. These double chains are linked by centrosymmetric pairs of O—H⋯O hydrogen bonds into layers parallel to (10-1). The asymmetric unit consists of one crystallographically independent Ni^II^ cation, one carboxyl­ate and one DFDI ligand, as well as of one water mol­ecule, all of them located in general positions.

## Related literature   

For the synthesis and structures of related Ni and naphthalenedi­carboxyl­ates, see: Guo *et al.* (2013 ▶); Kaduk & Hanko (2001 ▶).
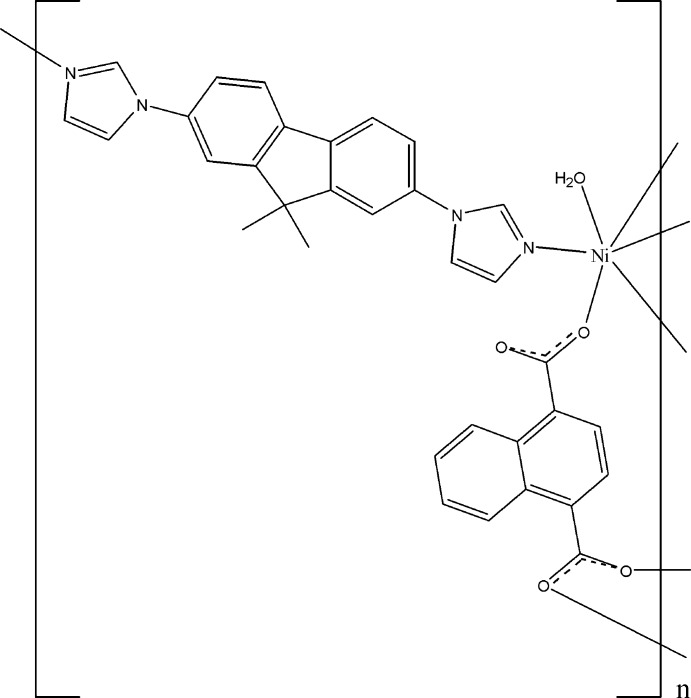


## Experimental   

### Crystal data  

[Ni(C_12_H_6_O_4_)(C_21_H_18_N_4_)(H_2_O)]
*M* *_r_* = 617.29
Monoclinic, 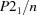

*a* = 11.1696 (17) Å
*b* = 16.161 (2) Å
*c* = 16.004 (2) Å
β = 93.458 (3)°
*V* = 2883.6 (7) Å^3^
*Z* = 4
Mo *K*α radiation
μ = 0.72 mm^−1^
*T* = 293 K
0.30 × 0.24 × 0.21 mm

### Data collection  

Bruker SMART APEXII CCD diffractometer
Absorption correction: multi-scan (*SADABS*; Bruker, 2002 ▶) *T*_min_ = 0.801, *T*_max_ = 0.865
18236 measured reflections
7043 independent reflections
4614 reflections with *I* > 2σ(*I*)
*R*_int_ = 0.053

### Refinement  

*R*[*F* ^2^ > 2σ(*F* ^2^)] = 0.058
*wR*(*F* ^2^) = 0.167
*S* = 1.00
7043 reflections
394 parameters
2 restraints
H atoms treated by a mixture of independent and constrained refinement
Δρ_max_ = 1.24 e Å^−3^
Δρ_min_ = −0.80 e Å^−3^

### 

Data collection: *APEX2* (Bruker, 2002 ▶); cell refinement: *SAINT* (Bruker, 2002 ▶); data reduction: *SAINT*; program(s) used to solve structure: *SHELXTL* (Sheldrick, 2008 ▶); program(s) used to refine structure: *SHELXL97* (Sheldrick, 2008 ▶); molecular graphics: *DIAMOND* (Brandenburg, 2008 ▶); software used to prepare material for publication: *SHELXTL* and *publCIF* (Westrip, 2010 ▶).

## Supplementary Material

Crystal structure: contains datablock(s) global, I. DOI: 10.1107/S1600536814017681/nc2327sup1.cif

Structure factors: contains datablock(s) I. DOI: 10.1107/S1600536814017681/nc2327Isup2.hkl

**Fig. 1.**
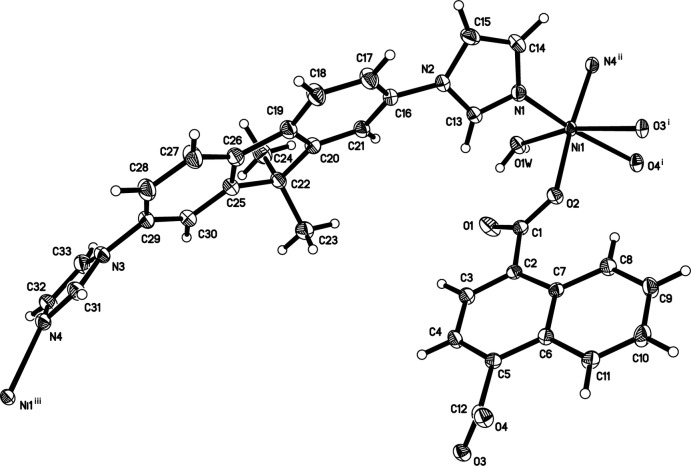
A view of the molecule of the title compound. Displacement ellipsoids are drawn at the 30% probability level. (i) -x + 1/2, y - 1/2, -z + 3/2; (ii) x, y - 1, z; (iii) x, y + 1, z.

**Fig. 2.**
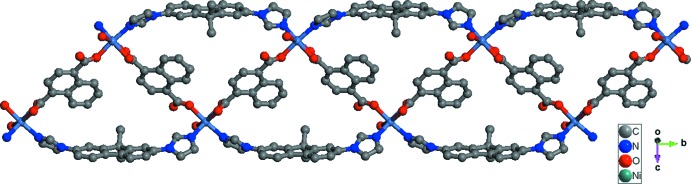
Crystal structure of the title compound with view along the a-axis. Hydrogen atoms are omitted for clarity.

Click here for additional data file.x y z x y z x y z . DOI: 10.1107/S1600536814017681/nc2327fig1.tif
A view of the mol­ecule of the title compound. Displacement ellipsoids are drawn at the 30% probability level. (i) −*x* + 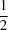, *y* − 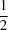, −*z* + 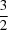; (ii) *x*, *y* − 1, *z*; (iii) *x*, *y* + 1, *z*.

a . DOI: 10.1107/S1600536814017681/nc2327fig2.tif
Crystal structure of the title compound with view along the *a*-axis. Hydrogen atoms are omitted for clarity.

CCDC reference: 1017498

Additional supporting information:  crystallographic information; 3D view; checkCIF report

## Figures and Tables

**Table 1:** Hydrogen-bond geometry (Å, °)

*D*—H⋯*A*	*D*—H	H⋯*A*	*D*⋯*A*	*D*—H⋯*A*
O1*W*—H1*A*⋯O1	0.85 (1)	1.88 (2)	2.659 (3)	152 (4)
O1*W*—H1*B*⋯O4^i^	0.85 (1)	1.94 (1)	2.791 (3)	176 (4)

Symmetry code: (i) 
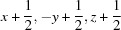
.

## supplementary crystallographic information

### S5. Synthesis and crystallization

The synthesis was performed under hydro­thermal conditions. A mixture of Ni(CH_3_COO)_2_^.^4(H_2_O), (0.2 mmol, 0.05 g), naphthalene-1,4-di­carb­oxy­lic acid (0.2 mmol, 0.044 g), 9,9-di­methyl-9H-fluorene-2,7-di­imidazole (0.2 mmol, 0.064 g) and H_2_O (15 mL) in a 25 mL stainless steel reactor with a Teflon liner was heated from 293 to 453 K in 2 h and a constant temperature was maintained at 453 K for 72 h, after which the mixture was cooled to 298 K. Green crystals of the title compound were recovered from the reaction.

### S6. Refinement

All C—H H atoms were positioned with idealized geometry and refined isotropic with *U*_iso_(H) = 1.2 *U*_eq_(C) (1.5 for methyl H atoms) using a riding model. The water H-atoms were located in a difference map and were refined with an O—H distance restrained to 0.85 (2) Å and with [*U*_iso_(H) = 1.5 *U*_eq_(O)].

### Crystal data

**Table:**

[Ni(C_12_H_6_O_4_)(C_21_H_18_N_4_)(H_2_O)]	*F*(000) = 1280
*M**_r_* = 617.29	*D*_x_ = 1.422 Mg m^−^^3^
Monoclinic, *P*2_1_/*n*	Mo *K*α radiation, λ = 0.71073 Å
Hall symbol: -P 2yn	Cell parameters from 7293 reflections
*a* = 11.1696 (17) Å	θ = 1.7–22.8°
*b* = 16.161 (2) Å	µ = 0.72 mm^−^^1^
*c* = 16.004 (2) Å	*T* = 293 K
β = 93.458 (3)°	Block, green
*V* = 2883.6 (7) Å^3^	0.30 × 0.24 × 0.21 mm
*Z* = 4	

### Data collection

**Table:**

Bruker SMART APEXII CCD diffractometer	7043 independent reflections
Radiation source: fine-focus sealed tube	4614 reflections with *I* > 2σ(*I*)
Graphite monochromator	*R*_int_ = 0.053
phi and ω scans	θ_max_ = 28.5°, θ_min_ = 2.2°
Absorption correction: multi-scan (*SADABS*; Bruker, 2002)	*h* = −14→10
*T*_min_ = 0.801, *T*_max_ = 0.865	*k* = −20→21
18236 measured reflections	*l* = −15→21

### Refinement

**Table:**

Refinement on *F*^2^	Primary atom site location: structure-invariant direct methods
Least-squares matrix: full	Secondary atom site location: difference Fourier map
*R*[*F*^2^ > 2σ(*F*^2^)] = 0.058	Hydrogen site location: inferred from neighbouring sites
*wR*(*F*^2^) = 0.167	H atoms treated by a mixture of independent and constrained refinement
*S* = 1.00	*w* = 1/[σ^2^(*F*_o_^2^) + (0.0959*P*)^2^] where *P* = (*F*_o_^2^ + 2*F*_c_^2^)/3
7043 reflections	(Δ/σ)_max_ = 0.001
394 parameters	Δρ_max_ = 1.24 e Å^−^^3^
2 restraints	Δρ_min_ = −0.80 e Å^−^^3^

### Special details

**Table:**

Geometry. All e.s.d.'s (except the e.s.d. in the dihedral angle between two l.s. planes) are estimated using the full covariance matrix. The cell e.s.d.'s are taken into account individually in the estimation of e.s.d.'s in distances, angles and torsion angles; correlations between e.s.d.'s in cell parameters are only used when they are defined by crystal symmetry. An approximate (isotropic) treatment of cell e.s.d.'s is used for estimating e.s.d.'s involving l.s. planes.
Refinement. Refinement of *F*^2^ against ALL reflections. The weighted *R*-factor *wR* and goodness of fit *S* are based on *F*^2^, conventional *R*-factors *R* are based on *F*, with *F* set to zero for negative *F*^2^. The threshold expression of *F*^2^ > σ(*F*^2^) is used only for calculating *R*-factors(gt) *etc*. and is not relevant to the choice of reflections for refinement. *R*-factors based on *F*^2^ are statistically about twice as large as those based on *F*, and *R*- factors based on ALL data will be even larger.

### Fractional atomic coordinates and isotropic or equivalent isotropic displacement parameters (Å^2^)

**Table:**

	*x*	*y*	*z*	*U*_iso_*/*U*_eq_	
Ni1	0.27771 (4)	0.06178 (2)	0.98863 (2)	0.02497 (14)	
C1	0.3429 (3)	0.21356 (19)	0.88921 (19)	0.0279 (7)	
C2	0.3086 (3)	0.27180 (18)	0.81633 (18)	0.0257 (7)	
C3	0.3433 (3)	0.35240 (19)	0.8250 (2)	0.0316 (7)	
H3	0.3853	0.3687	0.8742	0.038*	
C4	0.3180 (3)	0.4110 (2)	0.7628 (2)	0.0325 (8)	
H4	0.3430	0.4654	0.7712	0.039*	
C5	0.2566 (3)	0.38957 (19)	0.68914 (19)	0.0262 (7)	
C6	0.2184 (3)	0.30647 (18)	0.67658 (19)	0.0260 (7)	
C7	0.2414 (3)	0.24588 (18)	0.74100 (18)	0.0247 (6)	
C8	0.1991 (3)	0.1646 (2)	0.7261 (2)	0.0346 (8)	
H8	0.2118	0.1247	0.7676	0.042*	
C9	0.1399 (4)	0.1433 (2)	0.6520 (2)	0.0408 (9)	
H9	0.1115	0.0896	0.6440	0.049*	
C10	0.1217 (4)	0.2020 (2)	0.5879 (2)	0.0417 (9)	
H10	0.0828	0.1868	0.5372	0.050*	
C11	0.1606 (3)	0.2807 (2)	0.59974 (19)	0.0313 (7)	
H11	0.1491	0.3187	0.5564	0.038*	
C12	0.2384 (3)	0.45528 (18)	0.62262 (19)	0.0259 (7)	
C13	0.1741 (3)	0.2175 (2)	1.0487 (2)	0.0391 (9)	
H13	0.2352	0.2454	1.0236	0.047*	
C14	0.0603 (3)	0.1215 (2)	1.0900 (2)	0.0363 (8)	
H14	0.0281	0.0694	1.0989	0.044*	
C15	0.0164 (3)	0.1935 (2)	1.1183 (2)	0.0412 (9)	
H15	−0.0506	0.2000	1.1495	0.049*	
C16	0.0811 (3)	0.3425 (2)	1.1046 (2)	0.0321 (7)	
C17	−0.0300 (3)	0.3811 (2)	1.1055 (3)	0.0431 (9)	
H17	−0.1000	0.3499	1.0996	0.052*	
C18	−0.0362 (3)	0.4668 (2)	1.1155 (3)	0.0451 (10)	
H18	−0.1101	0.4930	1.1176	0.054*	
C19	0.0692 (3)	0.5122 (2)	1.1223 (2)	0.0362 (8)	
C20	0.1806 (3)	0.4719 (2)	1.1219 (2)	0.0316 (7)	
C21	0.1867 (3)	0.38737 (19)	1.1146 (2)	0.0328 (7)	
H21	0.2605	0.3606	1.1164	0.039*	
C22	0.2840 (3)	0.5320 (2)	1.1301 (2)	0.0322 (7)	
C23	0.3652 (4)	0.5239 (2)	1.0574 (3)	0.0451 (9)	
H23A	0.4298	0.5629	1.0643	0.068*	
H23B	0.3197	0.5350	1.0057	0.068*	
H23C	0.3971	0.4688	1.0562	0.068*	
C24	0.3560 (4)	0.5212 (2)	1.2141 (2)	0.0462 (9)	
H24A	0.4210	0.5601	1.2178	0.069*	
H24B	0.3873	0.4660	1.2181	0.069*	
H24C	0.3046	0.5308	1.2591	0.069*	
C25	0.2157 (3)	0.6146 (2)	1.1293 (2)	0.0349 (8)	
C26	0.0924 (3)	0.6021 (2)	1.1263 (2)	0.0365 (8)	
C27	0.0149 (4)	0.6698 (2)	1.1260 (3)	0.0477 (10)	
H27	−0.0676	0.6620	1.1253	0.057*	
C28	0.0631 (4)	0.7491 (2)	1.1265 (3)	0.0454 (10)	
H28	0.0127	0.7949	1.1263	0.054*	
C29	0.1852 (3)	0.7599 (2)	1.1273 (2)	0.0366 (8)	
C30	0.2639 (3)	0.69321 (19)	1.1287 (2)	0.0343 (8)	
H30	0.3463	0.7013	1.1293	0.041*	
C31	0.1982 (3)	0.9034 (2)	1.0717 (2)	0.0361 (8)	
H31	0.1278	0.9016	1.0376	0.043*	
C32	0.3630 (4)	0.9439 (2)	1.1328 (2)	0.0381 (8)	
H32	0.4286	0.9768	1.1491	0.046*	
C33	0.3425 (4)	0.8680 (2)	1.1618 (2)	0.0428 (9)	
H33	0.3912	0.8387	1.2005	0.051*	
N1	0.1599 (3)	0.13708 (16)	1.04611 (17)	0.0311 (6)	
N2	0.0896 (3)	0.25512 (16)	1.09216 (17)	0.0327 (6)	
N3	0.2357 (3)	0.84159 (16)	1.12350 (17)	0.0334 (7)	
N4	0.2723 (3)	0.96596 (16)	1.07513 (17)	0.0320 (6)	
O1	0.4258 (3)	0.23703 (16)	0.93909 (16)	0.0480 (7)	
O2	0.2836 (2)	0.14906 (14)	0.89537 (13)	0.0362 (6)	
O3	0.3289 (2)	0.49273 (13)	0.59960 (14)	0.0303 (5)	
O4	0.1345 (2)	0.47139 (14)	0.59113 (14)	0.0324 (5)	
O1W	0.4228 (2)	0.11528 (14)	1.05066 (15)	0.0347 (6)	
H1A	0.433 (4)	0.1628 (12)	1.030 (2)	0.052*	
H1B	0.488 (2)	0.089 (2)	1.060 (3)	0.052*	

### Atomic displacement parameters (Å^2^)

**Table:**

	*U*^11^	*U*^22^	*U*^33^	*U*^12^	*U*^13^	*U*^23^
Ni1	0.0363 (3)	0.0194 (2)	0.0184 (2)	−0.00295 (17)	−0.00517 (16)	−0.00024 (14)
C1	0.0347 (18)	0.0284 (16)	0.0200 (15)	−0.0009 (14)	−0.0045 (13)	0.0032 (12)
C2	0.0313 (17)	0.0266 (16)	0.0186 (15)	−0.0008 (13)	−0.0031 (12)	0.0037 (11)
C3	0.042 (2)	0.0285 (17)	0.0228 (16)	−0.0043 (14)	−0.0094 (14)	0.0027 (12)
C4	0.044 (2)	0.0213 (15)	0.0312 (18)	−0.0038 (14)	−0.0074 (15)	0.0014 (13)
C5	0.0298 (17)	0.0253 (16)	0.0229 (16)	0.0032 (13)	−0.0029 (13)	0.0031 (12)
C6	0.0298 (17)	0.0227 (15)	0.0248 (16)	0.0042 (12)	−0.0034 (13)	0.0018 (12)
C7	0.0281 (16)	0.0257 (15)	0.0201 (15)	0.0007 (13)	−0.0012 (12)	0.0011 (11)
C8	0.051 (2)	0.0281 (17)	0.0236 (17)	−0.0022 (15)	−0.0080 (15)	0.0046 (13)
C9	0.061 (3)	0.0276 (18)	0.0322 (19)	−0.0095 (16)	−0.0139 (17)	0.0017 (14)
C10	0.058 (2)	0.036 (2)	0.0280 (18)	0.0009 (17)	−0.0164 (17)	−0.0028 (14)
C11	0.0383 (19)	0.0304 (17)	0.0238 (16)	0.0065 (14)	−0.0080 (14)	0.0039 (13)
C12	0.0343 (18)	0.0199 (15)	0.0229 (16)	0.0050 (12)	−0.0033 (13)	−0.0019 (11)
C13	0.048 (2)	0.0261 (17)	0.045 (2)	−0.0079 (15)	0.0165 (17)	−0.0048 (15)
C14	0.0361 (19)	0.0251 (17)	0.047 (2)	−0.0066 (14)	0.0017 (16)	0.0033 (15)
C15	0.036 (2)	0.036 (2)	0.053 (2)	−0.0067 (16)	0.0144 (17)	0.0021 (16)
C16	0.0396 (19)	0.0251 (16)	0.0321 (18)	−0.0031 (14)	0.0069 (15)	−0.0033 (13)
C17	0.034 (2)	0.0336 (19)	0.062 (3)	−0.0042 (16)	0.0066 (18)	−0.0045 (17)
C18	0.033 (2)	0.0310 (19)	0.072 (3)	0.0013 (16)	0.0112 (19)	−0.0045 (18)
C19	0.038 (2)	0.0272 (18)	0.044 (2)	0.0015 (14)	0.0075 (16)	0.0007 (14)
C20	0.0365 (19)	0.0278 (16)	0.0306 (18)	−0.0030 (14)	0.0027 (14)	0.0002 (13)
C21	0.0341 (19)	0.0262 (17)	0.0387 (19)	0.0017 (14)	0.0062 (15)	0.0005 (13)
C22	0.0342 (19)	0.0253 (16)	0.0372 (19)	−0.0034 (14)	0.0034 (15)	0.0010 (13)
C23	0.048 (2)	0.038 (2)	0.051 (2)	−0.0016 (17)	0.0150 (19)	−0.0010 (17)
C24	0.045 (2)	0.042 (2)	0.051 (2)	−0.0063 (18)	−0.0086 (18)	0.0022 (17)
C25	0.041 (2)	0.0267 (17)	0.037 (2)	−0.0019 (15)	0.0054 (16)	0.0014 (14)
C26	0.037 (2)	0.0293 (18)	0.044 (2)	−0.0020 (15)	0.0080 (16)	−0.0014 (15)
C27	0.041 (2)	0.0310 (19)	0.072 (3)	0.0023 (16)	0.007 (2)	0.0005 (18)
C28	0.046 (2)	0.0292 (19)	0.062 (3)	0.0064 (17)	0.011 (2)	0.0000 (17)
C29	0.052 (2)	0.0249 (17)	0.0331 (19)	−0.0045 (15)	0.0048 (16)	0.0005 (13)
C30	0.039 (2)	0.0275 (17)	0.0363 (19)	−0.0031 (15)	0.0044 (15)	0.0022 (14)
C31	0.045 (2)	0.0282 (17)	0.0350 (19)	−0.0012 (15)	−0.0026 (16)	0.0047 (14)
C32	0.053 (2)	0.0317 (18)	0.0288 (18)	−0.0091 (16)	−0.0068 (16)	0.0026 (14)
C33	0.057 (2)	0.036 (2)	0.034 (2)	−0.0008 (18)	−0.0058 (18)	0.0041 (15)
N1	0.0364 (16)	0.0278 (14)	0.0286 (15)	−0.0027 (12)	−0.0017 (12)	−0.0020 (11)
N2	0.0374 (16)	0.0243 (14)	0.0367 (16)	−0.0040 (12)	0.0062 (13)	−0.0036 (11)
N3	0.0488 (18)	0.0229 (13)	0.0286 (15)	−0.0007 (12)	0.0026 (13)	0.0038 (11)
N4	0.0475 (18)	0.0246 (14)	0.0235 (14)	−0.0041 (13)	−0.0009 (12)	−0.0014 (11)
O1	0.0564 (17)	0.0449 (15)	0.0391 (15)	−0.0187 (13)	−0.0280 (12)	0.0196 (11)
O2	0.0568 (16)	0.0293 (12)	0.0210 (12)	−0.0104 (11)	−0.0115 (11)	0.0078 (9)
O3	0.0333 (13)	0.0252 (11)	0.0316 (13)	0.0023 (10)	−0.0049 (10)	0.0072 (9)
O4	0.0338 (13)	0.0294 (12)	0.0327 (13)	0.0021 (10)	−0.0084 (10)	0.0050 (9)
O1W	0.0399 (14)	0.0281 (13)	0.0346 (13)	−0.0050 (11)	−0.0107 (11)	0.0041 (10)

### Geometric parameters (Å, º)

**Table:**

Ni1—O1W	2.041 (2)	C17—C18	1.397 (5)
Ni1—N1	2.050 (3)	C17—H17	0.9300
Ni1—O2	2.058 (2)	C18—C19	1.387 (5)
Ni1—N4^i^	2.080 (3)	C18—H18	0.9300
Ni1—O3^ii^	2.111 (2)	C19—C20	1.405 (5)
Ni1—O4^ii^	2.208 (2)	C19—C26	1.477 (5)
Ni1—C12^ii^	2.475 (3)	C20—C21	1.373 (5)
C1—O2	1.242 (4)	C20—C22	1.509 (5)
C1—O1	1.245 (4)	C21—H21	0.9300
C1—C2	1.529 (4)	C22—C23	1.524 (5)
C2—C3	1.364 (4)	C22—C24	1.534 (5)
C2—C7	1.443 (4)	C22—C25	1.536 (5)
C3—C4	1.391 (4)	C23—H23A	0.9600
C3—H3	0.9300	C23—H23B	0.9600
C4—C5	1.372 (4)	C23—H23C	0.9600
C4—H4	0.9300	C24—H24A	0.9600
C5—C6	1.420 (4)	C24—H24B	0.9600
C5—C12	1.509 (4)	C24—H24C	0.9600
C6—C11	1.417 (4)	C25—C30	1.380 (4)
C6—C7	1.434 (4)	C25—C26	1.390 (5)
C7—C8	1.411 (4)	C26—C27	1.394 (5)
C8—C9	1.367 (5)	C27—C28	1.391 (5)
C8—H8	0.9300	C27—H27	0.9300
C9—C10	1.403 (5)	C28—C29	1.374 (5)
C9—H9	0.9300	C28—H28	0.9300
C10—C11	1.354 (5)	C29—C30	1.389 (5)
C10—H10	0.9300	C29—N3	1.439 (4)
C11—H11	0.9300	C30—H30	0.9300
C12—O3	1.253 (4)	C31—N4	1.306 (4)
C12—O4	1.264 (4)	C31—N3	1.348 (4)
C12—Ni1^iii^	2.475 (3)	C31—H31	0.9300
C13—N1	1.309 (4)	C32—C33	1.336 (5)
C13—N2	1.350 (4)	C32—N4	1.375 (5)
C13—H13	0.9300	C32—H32	0.9300
C14—C15	1.351 (5)	C33—N3	1.375 (5)
C14—N1	1.375 (4)	C33—H33	0.9300
C14—H14	0.9300	N4—Ni1^iv^	2.080 (3)
C15—N2	1.370 (4)	O3—Ni1^iii^	2.111 (2)
C15—H15	0.9300	O4—Ni1^iii^	2.208 (2)
C16—C21	1.386 (5)	O1W—H1A	0.848 (10)
C16—C17	1.390 (5)	O1W—H1B	0.851 (10)
C16—N2	1.430 (4)		
			
O1W—Ni1—N1	92.43 (11)	C19—C18—C17	119.1 (3)
O1W—Ni1—O2	90.18 (9)	C19—C18—H18	120.4
N1—Ni1—O2	88.17 (11)	C17—C18—H18	120.4
O1W—Ni1—N4^i^	92.62 (10)	C18—C19—C20	120.1 (3)
N1—Ni1—N4^i^	95.62 (11)	C18—C19—C26	132.1 (3)
O2—Ni1—N4^i^	175.18 (10)	C20—C19—C26	107.7 (3)
O1W—Ni1—O3^ii^	161.60 (10)	C21—C20—C19	120.7 (3)
N1—Ni1—O3^ii^	105.22 (10)	C21—C20—C22	127.3 (3)
O2—Ni1—O3^ii^	85.34 (9)	C19—C20—C22	111.9 (3)
N4^i^—Ni1—O3^ii^	90.79 (9)	C20—C21—C16	118.9 (3)
O1W—Ni1—O4^ii^	101.21 (9)	C20—C21—H21	120.5
N1—Ni1—O4^ii^	166.24 (10)	C16—C21—H21	120.5
O2—Ni1—O4^ii^	89.96 (9)	C20—C22—C23	111.5 (3)
N4^i^—Ni1—O4^ii^	85.64 (10)	C20—C22—C24	111.1 (3)
O3^ii^—Ni1—O4^ii^	61.03 (8)	C23—C22—C24	110.8 (3)
O1W—Ni1—C12^ii^	131.73 (11)	C20—C22—C25	100.5 (3)
N1—Ni1—C12^ii^	135.62 (11)	C23—C22—C25	112.7 (3)
O2—Ni1—C12^ii^	87.65 (9)	C24—C22—C25	109.9 (3)
N4^i^—Ni1—C12^ii^	87.55 (10)	C22—C23—H23A	109.5
O3^ii^—Ni1—C12^ii^	30.40 (10)	C22—C23—H23B	109.5
O4^ii^—Ni1—C12^ii^	30.64 (9)	H23A—C23—H23B	109.5
O2—C1—O1	125.7 (3)	C22—C23—H23C	109.5
O2—C1—C2	117.9 (3)	H23A—C23—H23C	109.5
O1—C1—C2	116.4 (3)	H23B—C23—H23C	109.5
C3—C2—C7	119.5 (3)	C22—C24—H24A	109.5
C3—C2—C1	117.0 (3)	C22—C24—H24B	109.5
C7—C2—C1	123.5 (3)	H24A—C24—H24B	109.5
C2—C3—C4	122.2 (3)	C22—C24—H24C	109.5
C2—C3—H3	118.9	H24A—C24—H24C	109.5
C4—C3—H3	118.9	H24B—C24—H24C	109.5
C5—C4—C3	120.8 (3)	C30—C25—C26	121.2 (3)
C5—C4—H4	119.6	C30—C25—C22	127.4 (3)
C3—C4—H4	119.6	C26—C25—C22	111.4 (3)
C4—C5—C6	119.4 (3)	C25—C26—C27	120.0 (3)
C4—C5—C12	117.8 (3)	C25—C26—C19	108.3 (3)
C6—C5—C12	122.7 (3)	C27—C26—C19	131.6 (3)
C11—C6—C5	121.2 (3)	C28—C27—C26	118.9 (4)
C11—C6—C7	118.4 (3)	C28—C27—H27	120.6
C5—C6—C7	120.3 (3)	C26—C27—H27	120.6
C8—C7—C6	118.1 (3)	C29—C28—C27	120.0 (3)
C8—C7—C2	124.3 (3)	C29—C28—H28	120.0
C6—C7—C2	117.6 (3)	C27—C28—H28	120.0
C9—C8—C7	121.4 (3)	C28—C29—C30	121.9 (3)
C9—C8—H8	119.3	C28—C29—N3	120.5 (3)
C7—C8—H8	119.3	C30—C29—N3	117.6 (3)
C8—C9—C10	120.3 (3)	C25—C30—C29	117.9 (3)
C8—C9—H9	119.8	C25—C30—H30	121.0
C10—C9—H9	119.8	C29—C30—H30	121.1
C11—C10—C9	120.2 (3)	N4—C31—N3	112.0 (3)
C11—C10—H10	119.9	N4—C31—H31	124.0
C9—C10—H10	119.9	N3—C31—H31	124.0
C10—C11—C6	121.6 (3)	C33—C32—N4	109.6 (3)
C10—C11—H11	119.2	C33—C32—H32	125.2
C6—C11—H11	119.2	N4—C32—H32	125.2
O3—C12—O4	121.4 (3)	C32—C33—N3	107.1 (3)
O3—C12—C5	118.1 (3)	C32—C33—H33	126.5
O4—C12—C5	120.5 (3)	N3—C33—H33	126.5
O3—C12—Ni1^iii^	58.50 (15)	C13—N1—C14	105.4 (3)
O4—C12—Ni1^iii^	62.91 (16)	C13—N1—Ni1	121.6 (2)
C5—C12—Ni1^iii^	176.3 (2)	C14—N1—Ni1	133.0 (2)
N1—C13—N2	112.1 (3)	C13—N2—C15	106.2 (3)
N1—C13—H13	123.9	C13—N2—C16	124.8 (3)
N2—C13—H13	123.9	C15—N2—C16	129.0 (3)
C15—C14—N1	109.6 (3)	C31—N3—C33	105.8 (3)
C15—C14—H14	125.2	C31—N3—C29	126.7 (3)
N1—C14—H14	125.2	C33—N3—C29	126.7 (3)
C14—C15—N2	106.7 (3)	C31—N4—C32	105.5 (3)
C14—C15—H15	126.6	C31—N4—Ni1^iv^	126.3 (2)
N2—C15—H15	126.6	C32—N4—Ni1^iv^	126.1 (2)
C21—C16—C17	121.2 (3)	C1—O2—Ni1	132.4 (2)
C21—C16—N2	118.0 (3)	C12—O3—Ni1^iii^	91.11 (18)
C17—C16—N2	120.8 (3)	C12—O4—Ni1^iii^	86.45 (19)
C16—C17—C18	119.8 (3)	Ni1—O1W—H1A	108 (3)
C16—C17—H17	120.1	Ni1—O1W—H1B	122 (3)
C18—C17—H17	120.1	H1A—O1W—H1B	113 (4)
			
O2—C1—C2—C3	158.1 (3)	C22—C25—C26—C19	−1.8 (4)
O1—C1—C2—C3	−19.3 (5)	C18—C19—C26—C25	−177.5 (4)
O2—C1—C2—C7	−21.1 (5)	C20—C19—C26—C25	−0.7 (4)
O1—C1—C2—C7	161.4 (3)	C18—C19—C26—C27	1.2 (7)
C7—C2—C3—C4	−1.2 (5)	C20—C19—C26—C27	178.0 (4)
C1—C2—C3—C4	179.6 (3)	C25—C26—C27—C28	1.5 (6)
C2—C3—C4—C5	−0.2 (6)	C19—C26—C27—C28	−177.0 (4)
C3—C4—C5—C6	0.1 (5)	C26—C27—C28—C29	0.1 (6)
C3—C4—C5—C12	−176.7 (3)	C27—C28—C29—C30	−0.9 (6)
C4—C5—C6—C11	−176.5 (3)	C27—C28—C29—N3	176.8 (4)
C12—C5—C6—C11	0.1 (5)	C26—C25—C30—C29	1.6 (5)
C4—C5—C6—C7	1.4 (5)	C22—C25—C30—C29	179.5 (3)
C12—C5—C6—C7	178.0 (3)	C28—C29—C30—C25	0.0 (5)
C11—C6—C7—C8	−3.5 (5)	N3—C29—C30—C25	−177.7 (3)
C5—C6—C7—C8	178.5 (3)	N4—C32—C33—N3	−1.3 (4)
C11—C6—C7—C2	175.3 (3)	N2—C13—N1—C14	0.2 (4)
C5—C6—C7—C2	−2.7 (5)	N2—C13—N1—Ni1	−177.6 (2)
C3—C2—C7—C8	−178.7 (3)	C15—C14—N1—C13	0.0 (4)
C1—C2—C7—C8	0.5 (5)	C15—C14—N1—Ni1	177.5 (3)
C3—C2—C7—C6	2.5 (5)	O1W—Ni1—N1—C13	49.8 (3)
C1—C2—C7—C6	−178.3 (3)	O2—Ni1—N1—C13	−40.3 (3)
C6—C7—C8—C9	1.3 (5)	N4^i^—Ni1—N1—C13	142.7 (3)
C2—C7—C8—C9	−177.4 (3)	O3^ii^—Ni1—N1—C13	−124.9 (3)
C7—C8—C9—C10	1.2 (6)	O4^ii^—Ni1—N1—C13	−122.6 (4)
C8—C9—C10—C11	−1.3 (6)	C12^ii^—Ni1—N1—C13	−125.0 (3)
C9—C10—C11—C6	−1.1 (6)	O1W—Ni1—N1—C14	−127.3 (3)
C5—C6—C11—C10	−178.5 (3)	O2—Ni1—N1—C14	142.6 (3)
C7—C6—C11—C10	3.5 (5)	N4^i^—Ni1—N1—C14	−34.5 (3)
C4—C5—C12—O3	53.8 (4)	O3^ii^—Ni1—N1—C14	57.9 (3)
C6—C5—C12—O3	−122.8 (3)	O4^ii^—Ni1—N1—C14	60.2 (6)
C4—C5—C12—O4	−126.5 (3)	C12^ii^—Ni1—N1—C14	57.8 (4)
C6—C5—C12—O4	56.9 (4)	N1—C13—N2—C15	−0.4 (4)
C4—C5—C12—Ni1^iii^	77 (3)	N1—C13—N2—C16	−179.1 (3)
C6—C5—C12—Ni1^iii^	−100 (3)	C14—C15—N2—C13	0.4 (4)
N1—C14—C15—N2	−0.3 (4)	C14—C15—N2—C16	179.0 (3)
C21—C16—C17—C18	1.0 (6)	C21—C16—N2—C13	−35.6 (5)
N2—C16—C17—C18	−178.3 (3)	C17—C16—N2—C13	143.7 (4)
C16—C17—C18—C19	1.6 (6)	C21—C16—N2—C15	146.0 (4)
C17—C18—C19—C20	−2.2 (6)	C17—C16—N2—C15	−34.7 (6)
C17—C18—C19—C26	174.3 (4)	N4—C31—N3—C33	−0.4 (4)
C18—C19—C20—C21	0.3 (5)	N4—C31—N3—C29	−171.0 (3)
C26—C19—C20—C21	−177.0 (3)	C32—C33—N3—C31	1.0 (4)
C18—C19—C20—C22	−179.8 (3)	C32—C33—N3—C29	171.6 (3)
C26—C19—C20—C22	2.9 (4)	C28—C29—N3—C31	−45.2 (5)
C19—C20—C21—C16	2.3 (5)	C30—C29—N3—C31	132.6 (4)
C22—C20—C21—C16	−177.6 (3)	C28—C29—N3—C33	146.1 (4)
C17—C16—C21—C20	−2.9 (5)	C30—C29—N3—C33	−36.2 (5)
N2—C16—C21—C20	176.4 (3)	N3—C31—N4—C32	−0.4 (4)
C21—C20—C22—C23	56.6 (5)	N3—C31—N4—Ni1^iv^	163.9 (2)
C19—C20—C22—C23	−123.3 (3)	C33—C32—N4—C31	1.0 (4)
C21—C20—C22—C24	−67.5 (4)	C33—C32—N4—Ni1^iv^	−163.3 (3)
C19—C20—C22—C24	112.6 (3)	O1—C1—O2—Ni1	7.5 (6)
C21—C20—C22—C25	176.2 (3)	C2—C1—O2—Ni1	−169.7 (2)
C19—C20—C22—C25	−3.7 (4)	O1W—Ni1—O2—C1	−8.8 (3)
C20—C22—C25—C30	−174.8 (3)	N1—Ni1—O2—C1	83.7 (3)
C23—C22—C25—C30	−56.0 (5)	N4^i^—Ni1—O2—C1	−134.4 (12)
C24—C22—C25—C30	68.0 (4)	O3^ii^—Ni1—O2—C1	−170.9 (3)
C20—C22—C25—C26	3.3 (4)	O4^ii^—Ni1—O2—C1	−110.0 (3)
C23—C22—C25—C26	122.0 (3)	C12^ii^—Ni1—O2—C1	−140.5 (3)
C24—C22—C25—C26	−113.9 (3)	O4—C12—O3—Ni1^iii^	−1.4 (3)
C30—C25—C26—C27	−2.4 (6)	C5—C12—O3—Ni1^iii^	178.3 (2)
C22—C25—C26—C27	179.4 (3)	O3—C12—O4—Ni1^iii^	1.3 (3)
C30—C25—C26—C19	176.4 (3)	C5—C12—O4—Ni1^iii^	−178.4 (3)

Symmetry codes: (i) *x*, *y*−1, *z*; (ii) −*x*+1/2, *y*−1/2, −*z*+3/2; (iii) −*x*+1/2, *y*+1/2, −*z*+3/2; (iv) *x*, *y*+1, *z*.

### Hydrogen-bond geometry (Å, º)

**Table:**

*D*—H···*A*	*D*—H	H···*A*	*D*···*A*	*D*—H···*A*
O1*W*—H1*A*···O1	0.85 (1)	1.88 (2)	2.659 (3)	152 (4)
O1*W*—H1*B*···O4^v^	0.85 (1)	1.94 (1)	2.791 (3)	176 (4)

Symmetry code: (v) *x*+1/2, −*y*+1/2, *z*+1/2.
