# Supplementary figures and images for: Crystal structure of poly[aqua­[μ-1,1′-(9,9-dimethyl-9H-fluoren-2,7-di­yl)di-1H-imidazole](μ-naphthalene-1,4-di­carboxyl­ato)nickel(II)]
Source: Acta Crystallogr Sect E Struct Rep Online. 2014 Aug 6;70(Pt 9):m324–5. doi: 10.1107/S1600536814017681 (PMC4186126; doi:10.1107/S1600536814017681)

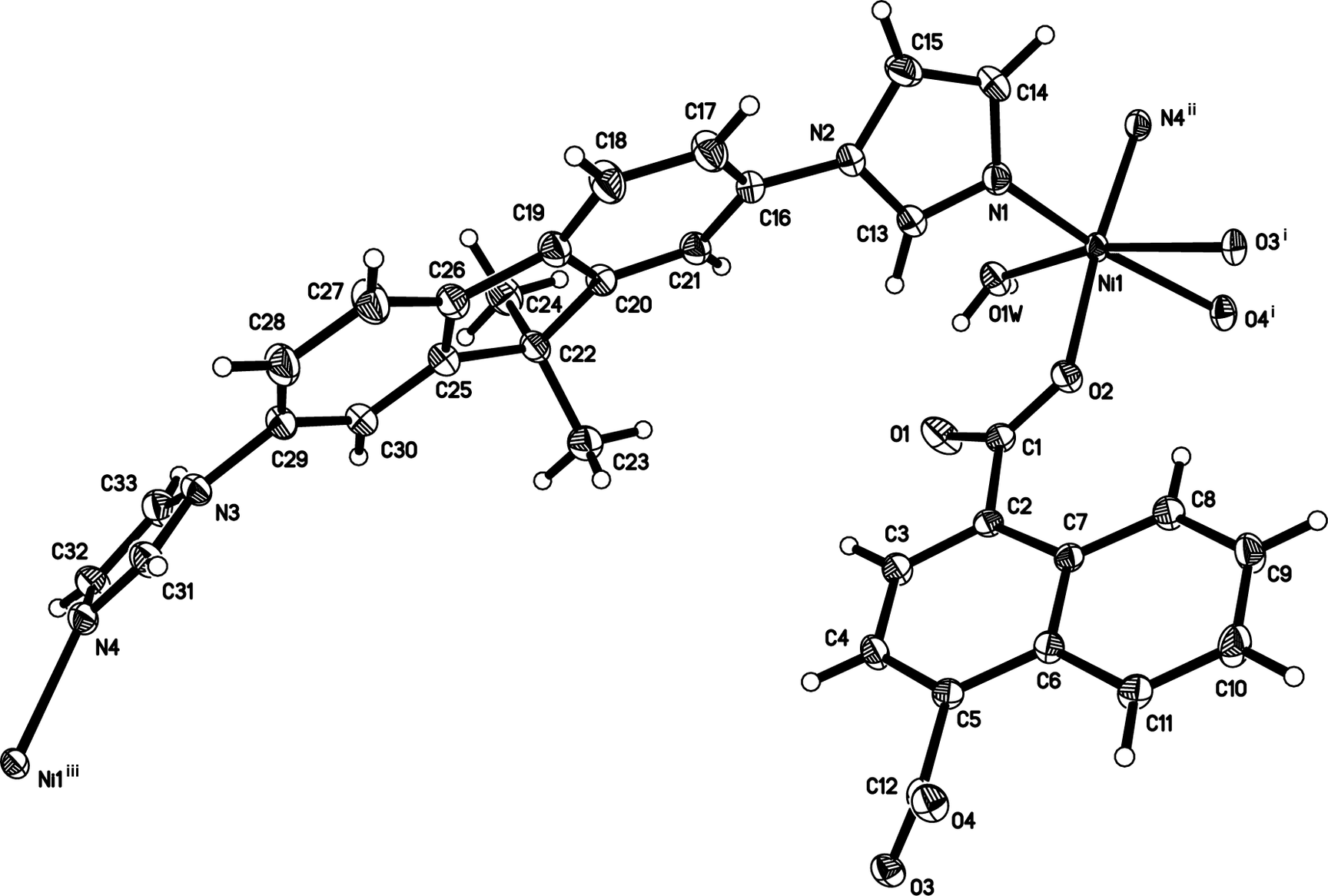

Supplement: Supplementary file 3 [file e-70-0m324-fig1.tif]

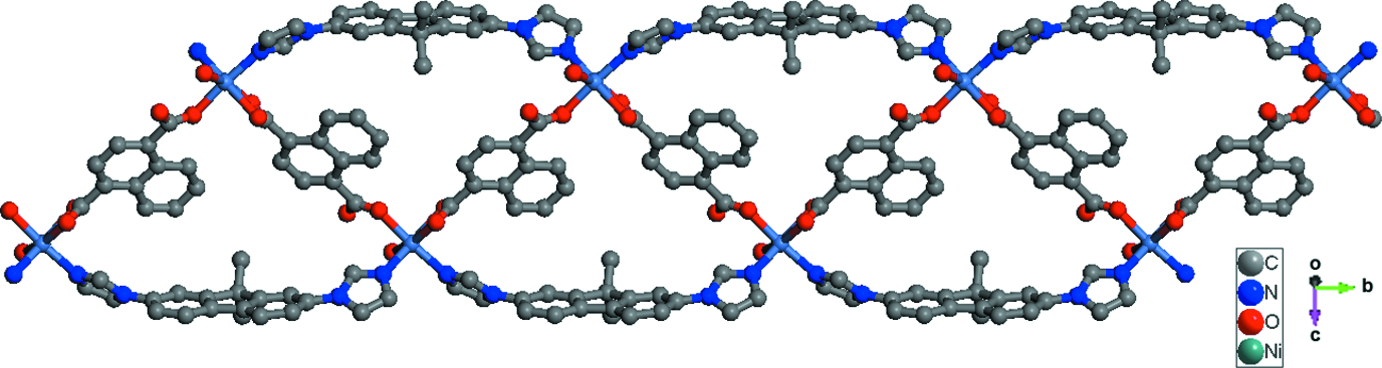

Supplement: Supplementary file 4 [file e-70-0m324-fig2.tif]
